# Exploring the Application of the Artificial-Intelligence-Integrated Platform 3D Slicer in Medical Imaging Education

**DOI:** 10.3390/diagnostics14020146

**Published:** 2024-01-08

**Authors:** Ying Zhang, Hongbo Feng, Yan Zhao, Shuo Zhang

**Affiliations:** 1 Second Department of Arrhythmia, Dalian Municipal Central Hospital Affiliated to Dalian University of Technology, Dalian 116089, China; 2 Department of Nuclear Medicine, The First Affiliated Hospital of Dalian Medical University, Dalian 116011, China; fenghongbo1@firsthosp-dmu.com; 3 Department of Information Center, The First Affiliated Hospital of Dalian Medical University, Dalian 116011, China

**Keywords:** 3D Slicer, medical imaging, open-source software, artifact intelligence (AI), medical education

## Abstract

Artificial Intelligence (AI) has revolutionized medical imaging procedures, specifically with regard to image segmentation, reconstruction, interpretation, and research. 3D Slicer, an open-source medical image analysis platform, has become a valuable tool in medical imaging education due to its integration of various AI applications. Through its open-source architecture, students can gain practical experience with diverse medical images and the latest AI technology, reinforcing their understanding of anatomy and imaging technology while fostering independent learning and clinical reasoning skills. The implementation of this platform improves instruction quality and nurtures skilled professionals who can meet the demands of clinical practice, research institutions, and technology innovation enterprises. AI algorithms’ application in medical image processing have facilitated their translation from the lab to practical clinical applications and education.

## 1. Introduction

The application of AI technologies, such as ChatGPT, has brought the immense potential of AI to the forefront, particularly in relation to medical imaging. AI technologies have found widespread applications in segmentation, reconstruction, and diagnosis, leading to a need for enhanced proficiency among medical imaging professionals and a reevaluation of medical imaging education.

Medical imaging is a complex field that relies on the integration of multiple disciplines to generate precise and high-resolution diagnostic images. Professionals in this field possess comprehensive expertise in various domains such as medicine, physics, engineering, and computer science. This extensive knowledge allows them to effectively analyze and interpret medical images, develop cutting-edge technologies, and enhance the functionality of advanced imaging equipment. With the continuous advancements in information and computer technology [1,2,3,4], medical imaging has become a rapidly evolving and extensively utilized discipline in modern medicine. To ensure that students are adequately prepared to utilize AI in their practices, it is essential to incorporate AI technologies and computer science knowledge into curricula.

Introducing courses focused on AI applications in medical imaging, machine learning algorithms, and data analysis techniques is one approach to achieving the aforementioned goal. By equipping students with the necessary knowledge and skills in these areas, they can effectively utilize AI technologies in their practices. Additionally, traditional teaching methods should be reevaluated and modernized to meet to the needs of AI-oriented radiologists and technologists. Integrating interactive learning techniques, such as case-based learning and virtual simulations, can enhance students’ comprehension and application of AI in medical imaging. Moreover, collaboration between medical and computer science faculties is essential for successfully incorporating AI into curricula. Joint workshops and interdisciplinary research projects can foster a better understanding of both fields and encourage innovation in AI-based medical imaging. Currently, there are two main approaches to medical imaging education: multimedia lecture-based learning (LBL) and problem-based learning (PBL) [5]. LBL involves educators using various media, such as pictures, videos, and virtual reality (VR), to convey imaging data to students. This approach aims to provide students with a clear understanding of different image types. On the other hand, PBL focuses on facilitating student exploration and discovery through questioning and discussion. While both approaches have their strengths and limitations, neither effectively empowers students to take initiative and establish a connection with the images [6]. An image tool with comprehensive functionality is urgently required to address this issue.

3D Slicer 5.6.1 is an open-source software for medical image processing and visualization used for medical image analysis and research (http://www.slicer.org, accessed on 12 December 2023), as shown in Table 1. It is a powerful platform for processing and visualizing multi-modal medical image data, such as those obtained via computed tomography (CT), magnetic resonance imaging (MRI), positron emission tomography (PET), etc. 3D Slicer is designed to provide a flexible, powerful, user-friendly AI-integrated platform for medical image processing and visualization. It has become one of the most popular and widely used software products in the field of medical image processing.

Despite many research studies reporting on the applications of 3D Slicer in the field of clinical medical imaging, there is a lack of reports on its use in medical image education. Determining effective strategies for utilizing new software in medical imaging education is an important issue. Therefore, further research is needed on how new platforms like 3D Slicer can enhance medical image education. This study is centered on exploring the potential value of using the 3D Slicer platform as a teaching tool in medical imaging education. The Research Questions (RQs) will guide this study:

- RQ 1: What are the applications of 3D Slicer in the field of clinical medical imaging?
- RQ 2: What are the potential applications of the 3D Slicer platform in medical imaging education?
- RQ 3: What key limitations are militating against the inclusion of the 3D Slicer platform in medical imaging education?

**Figure 1:**
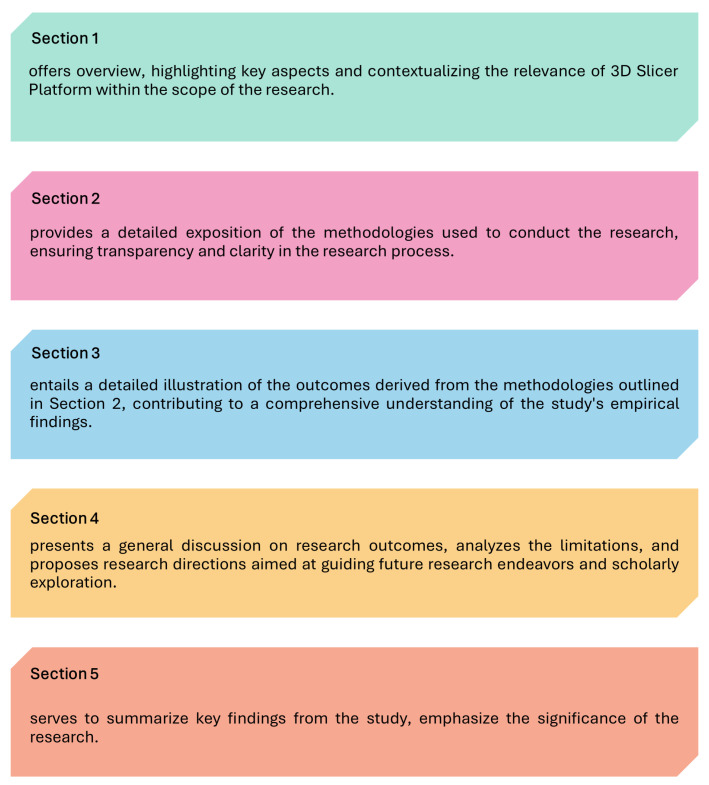
Flow chart outlining the key components of this article.

In this investigation, we conducted a non-systematic literature review, focusing on publications related to the application of the AI-integrated platform 3D Slicer in the fields of medicine and education. We delved into the potential utility of this platform in medical imaging education and explored the challenges it faces. The findings of the literature review were thematically organized, with a specific emphasis on the diverse applications of 3D Slicer in the educational domain. This article is organized as shown in Figure 1.

## 2. Methodology

The methodological approach employed in this non-systematic literature review encompasses a comprehensive examination of the predominant theories and research contributions published within the last 5 years on the specified topic. The foundation of this review rests on databases containing peer-reviewed content, including meta-analyses and review papers. The inquiry was initiated with a thorough exploration of the PubMed database, distinguished for its indexing of diverse online journals in the expansive field of biomedicine with a global perspective, facilitating the compilation of peer-reviewed academic papers. Additionally, a meticulous and exhaustive search was conducted on Web of Science, integrating pertinent keywords and studies extracted from the reference lists of peer-reviewed papers.

In terms of the search strategy employed, the initial investigation centered on probing the applications of 3D Slicer in the domains of medicine and education, utilizing specific search queries, namely, “applications” AND “3D Slicer” AND “medical”, “applications” AND “3D Slicer” AND “education”. The subsequent refinement of the search results involved the inclusion of terms such as “meta-analysis” and “review” or adjustments to search options based on specific features of individual databases. The inclusion and exclusion criteria that were considered in this study are presented in Table 2.

To ensure the reliability of the information extraction process and facilitate research synthesis, key characteristics of interest (specific applications) were identified, and tables were constructed accordingly. The methodology involved the principal investigator providing an initial overview of the synthesis, delineating the processes of coding, data entry, and data management. Coders, while adhering to a standardized set of instructions, operated independently. Findings were collated, and any disparities were identified and resolved. Additionally, to enhance the efficiency of the coding process, regular group meetings were conducted among all coders to discuss potential additions, deletions, and modifications. Considering the methodological diversity of the scrutinized documents, the synthesis of the literature review findings commenced based on comparable study designs. Ultimately, data retrieved from diverse sources were amalgamated across categories.

## 3. Findings

The research findings have established the application of 3D Slicer as an important medical image analysis platform in the medical field, highlighting its potential for application in medical imaging education, such as image segmentation and reconstruction, computer-aided diagnosis and research, and the quantitative analysis of medical imaging.

### 3.1. Application of 3D Slicer Platform in Medical Image Analysis

**Figure 2:**
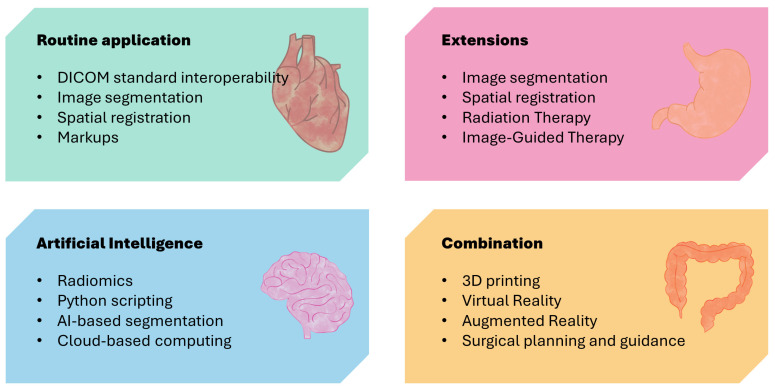
Chart of 3D Slicer approaches and applications.

The 3D Slicer open-source medical imaging analysis platform [7] features an advanced interactive and visualization interface capable of accommodating a multitude of medical image formats, including digital imaging and communications in medicine (DICOM), neuroimaging informatics technology initiative (NIfTI), and nearly raw raster data (NRRD) (Figure 2). It extracts pertinent information from medical images and transforms them into 3D models, generating multi-dimensional image data [8]. Beyond visualization, the platform is equipped to perform a series of operations, such as image processing, segmentation, registration, analysis, and quantitative analysis [9]. Moreover, it supports extensions, with over a hundred open-source extensions available on the platform. These extensions range from radiomics analysis [10,11] to artificial intelligence (AI)-based automatic organ segmentation for medical image analysis [12,13] and from surgical navigation [14,15] to target delineation [16,17] and dose calculation for radiation therapy clinical tools [18,19]. Its extensive functionality surpasses that of professional workstations utilized in clinical environments [20,21,22,23,24]. Table 3 presents the general applications of 17 studies examining the use of 3D Slicer as a medical image analysis platform.

Sareen et al. and Pujol et al. initiated explorations of the integration of image analysis software into anatomy and imaging education [25,26], yielding significant outcomes in domains such as anatomy and image interpretation. In recent years, the degree of informatization in hospitals has steadily advanced, with a concomitant escalation in demand for sophisticated analysis functions such as the quantitative analysis [27,28], fusion registration [29,30,31], and omics analysis [32,33,34] of images. These capabilities elude traditional instructional methods, resulting in a widening chasm between pedagogy and the exigencies of clinical practice and research.

**Table 3:** Summary of 3D Slicer as a medical image analysis platform and the implications of this finding for medical imaging education.

Author	Reference	Raw Data	Specific Application	Implication
Yuan et al., 2021	[35]	CT, MRI, 3D models, and the original image	Transforming 2D information from various imaging techniques into 3D information.	Optimizes medical-imaging-teaching activities for the development of modern medical education.
Hadi et al., 2022	[36]	SPECT and CT	Image segmentation, registration, and visualization.	Assists students in navigating intricate steps required in the importation of tomographic data.
Durnea et al., 2021	[21]	MRI	Creating 3D models base on cross-sectional image sets.	Provides a comprehensive and 3D display of anatomical structures and lesions.
Bindschadler et al., 2022	[37]	CT	Turning 4D Cardiac CT into VR/AR	4D imaging and related workflows will be useful for educational purposes.
Cao et al., 2022	[38]	CT	Calculating the surface area (S) and volume (V) of hematoma.	Providing quantitative indicators for student clinical practice.
Levine et al., 2020	[39]	CT and X-ray	Computationally simulating an X-ray-type image from CT data.	Provides an easy way for students to make digitally reconstructed radiographs
Yang et al., 2019	[40]	MRI	Aiding neurosurgeons in planning the location of an anticipated craniotomy relative to a preoperatively imaged tumor in a physical-to-virtual setup.	Provides more intuitive and interactive teaching tools.
Eskandari et al., 2020	[41]	CT	Assessing the irradiated volumes and both displacement magnitudes and vectors for the heart and left lung.	Enables assessment of the irradiation field in radiographic examination and estimation of radiation dose.
You et al., 2022	[42]	CT	Transforming medical images into digital models to prepare for 3D printing.	Compensates for the lack of skills in 3D design and modeling software.
Chen et al., 2022	[43]	CT	Automatically measuring the morphological parameters of the distal femur.	Enables automatic measurement of bones and other body parts.
Shi et al., 2022	[44]	MRI	Determining the preoperative evaluation value before microsurgical vascular decompression.	More accurate than MRI in preoperative evaluation of neurovascular relationship and offending vessel.
Liao et al., 2022	[45]	CT	Assisting endoscopic treatment for patients with hypertensive cerebral hemorrhage.	Better demonstrates and explains pathological changes and anatomical structures
Huie et al., 2022	[46]	CT	Iterating slice-by-slice through 3D structures to calculate second moment of area and other cross-sectional properties.	Analyzing the internal geometric structure of biological structures provides more possibilities.
Huang et al., 2022	[47]	CT	A fly-through visualization of both the inside and outside of the duct.	Provides more insight into pancreatic diseases than a fly-through visualization.
Briend et al., 2020	[48]	MRI	Medical image visualization and analysis.	Allows medical imaging to be presented to students in a more intuitive and clear manner.
Zaffino et al., 2020	[49]	CT	Providing and a basic input–output mechanism.	Providing diverse learning resources for medical students.
Liu et al., 2022	[50]	PET, MRI, and CT	Predicting epileptic lesions according to signals and morphology of images	Medical students can gain a deeper understanding of the characteristics and manifestations of epilepsy.

### 3.2. Potential Applications of the 3D Slicer Platform in Medical Imaging Education

As illustrated in Table 4, compared to traditional teaching methods, this platform demonstrates great potential in medical imaging education. In contrast to traditional commercial software, which necessitates exorbitant acquisition costs and specialized hardware, this open-source platform eliminates the need for purchasing licenses or installing specific hardware. This platform offers image examples, which can be coupled with the abundance of open-source image data available online.

With the aid of relevant documentation and tutorials, educators can more effectively organize and plan their lessons. Throughout the learning process, educators supply anonymized image examples or project files, offering students the opportunity to independently conduct image readings, generate corresponding 3D images, and even construct intricate models on their personal computers. By presenting thought-provoking questions or projects to be resolved, educators can fully engage students’ enthusiasm, encouraging them to actively seek solutions and solve problems. Students possessing programming skills can further develop and optimize the software to meet specific needs or create custom application extensions to improve their practical and innovative abilities [35]. This approach aligns with the philosophy of integrating medical imaging education theory with practice, rendering images visible, tangible, and functional.

#### 3.2.1. Application of 3D Slicer in Image Segmentation and Reconstruction

Hadi et al. [36] and Bindschadler et al. [37] reported that 3D Slicer can be a powerful tool for segmentation and reconstruction. Medical images often contain complex and changeable details and structures. Through image segmentation techniques, different tissues, organs, or lesion areas can be effectively separated and labeled in an image, providing clearer visual information. This is of great significance for helping medical students to learn and understand anatomical structures and disease characteristics. In addition, image reconstruction techniques can improve the quality and clarity of medical images by processing and restoring them. This is particularly helpful in teaching students to demonstrate subtle structures, hidden lesions, or low-contrast areas. Through image reconstruction, medical students can better observe and cognize the details in images, deepening their understanding of diseases.

**Figure 3:**
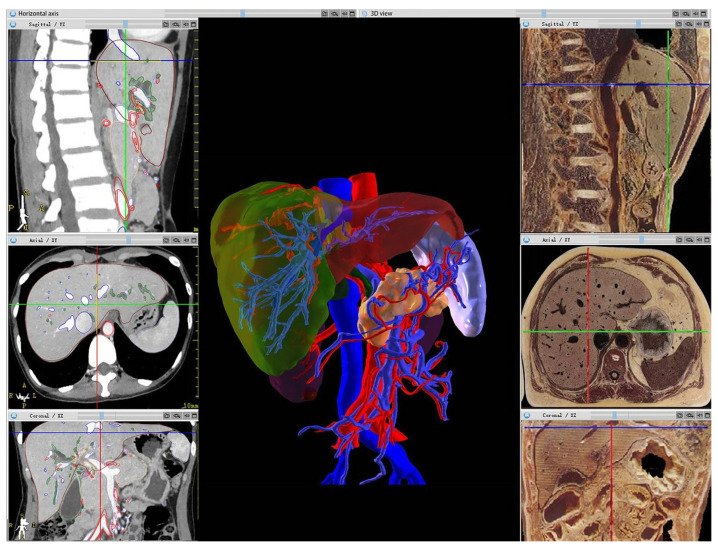
A liver CT scan is presented alongside its corresponding sectional anatomy. On the left side, we observe the liver CT image, while on the right side, we find a sectional anatomy representation. In the middle panel, a 3D reconstruction of the liver and its blood vessels, achieved through CT scanning, is presented [35].

Furthermore, by integrating visual technologies such as AR, VR, or 3D printing, anatomical structures and organ images can be vividly displayed without reliance on linguistic descriptions or students’ imagination (Figure 3). The traditional teaching method involves using specimens combined with pictures. While specimens are intuitive, they also pose many problems such as insufficient quantity. Pure picture displays lack important information such as 3D organizational relationships. Using integrated AI function modules such as TotalSegmentator, NvidiaAIAssited Annotation, and MONAILabel [51] to automatically segment images and obtain tissue labels helps to improve students’ capacity for independent learning, allowing them to understand the essential aspects of imaging. Teachers can focus more on designing scientific questions, fully mobilize students’ enthusiasm, and let them discover problems, explore solutions, and enhance their skills.

#### 3.2.2. Application of 3D Slicer in Computer-Aided Diagnosis and Research

Yuan et al. [35] reported the application of 3D Slicer in writing medical imaging reports. Writing medical imaging diagnostic reports is a basic skill for students majoring in medical imaging, which is also a challenging aspect for students. Computer-aided diagnosis systems, powered by AI, have proven to be invaluable tools for assisting healthcare professionals in accurately diagnosing various medical conditions. By analyzing medical images with advanced algorithms, AI can identify patterns and abnormalities that may not be easily detectable by the human eye. This not only enhances the accuracy of diagnoses but also helps in the early detection of diseases, leading to timely interventions and improved patient outcomes. 3D Slicer provides great assistance to medical students in writing medical imaging reports (Table 5).

Image processing plays a vital role in various research tasks, including clinical imaging. However, the high cost and lack of customizability of clinical imaging workstations pose significant challenges. Moreover, these workstations often come with limited functions and usage restrictions. In contrast, 3D Slicer offers numerous advantages. Being open source, 3D Slicer is a cross-platform software platform written in Python, requiring minimal hardware resources. It provides a wide range of applications through its extension store, offering nearly 200 extensions that cover diverse fields like deep learning, image segmentation, and simulation navigation. This rich variety of applications enhances its usability and versatility. Another notable advantage of 3D Slicer is its high level of customizability. Users can develop custom programs tailored to their research objectives and analysis needs. This flexibility allows researchers to explore and implement innovative approaches in their work.

#### 3.2.3. Application of 3D Slicer in Quantitative Analysis of Medical Imaging

**Figure 4:**
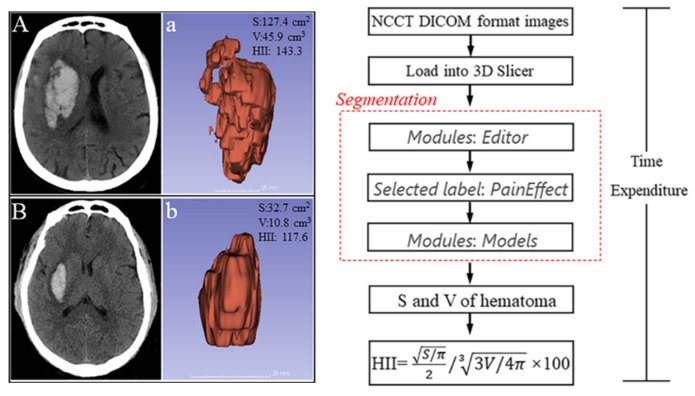
3D Slicer was employed to create a 3D representation of hematomas in two patients. In (**A**,**a**), an example of a patient with hematoma expansion is shown, while in (**B**,**b**), an example of a patient without hematoma expansion is shown. The original images are represented by (**A**,**B**), while the 3D model for the hematoma is represented by (**a**,**b**). The surface area is denoted as S, the volume is denoted as V, and the hematoma irregularity index is indicated by HII. The workflow for calculating the HII is described in detail. The DICOM image data were transferred to 3D Slicer, and the hematomas were identified semi-automatically pixel by pixel in each slice using the PaintEffect tool in the Editor module. The Models module in the software is then used to reconstruct a 3D representation by summing the volume of all the pixels. The software provides direct measurements of the volumes and surface area of the reconstructed hematoma [38].

Cao et al. reported the 3D-Slicer-based calculation of the hematoma irregularity index for predicting hematoma expansion in an intracerebral hemorrhage [38]. With the advancement of imaging technology and computer technology, traditional image reading has been unable to keep up with the progress of medical imaging technology, especially in some scientific research tasks where quantitative analysis has been assigned top priority. Quantitative analysis modules such as slicerDMRI and DSCMRI Analysis can help students gain a deep understanding of the application value of image acquisition methods, contrast-enhanced scanning, and magnetic resonance diffusion sequences, laying a solid foundation for their subsequent learning and work (Figure 4).

**Figure 5:**
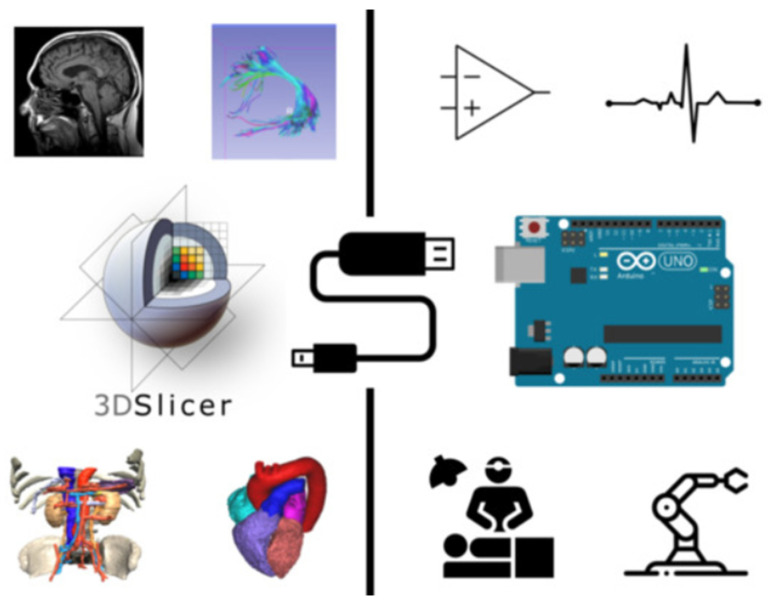
The extension applications of 3D Slicer involve outputting data from 3D Slicer to achieve integration between 3D Slicer and Arduino. It is suitable for rapid prototyping, basic input–output interactions, and educational purposes [49].

Zaffino et al. [49] reported that 3D Slicer can serve as a bridge between a medical imaging platform and a microcontroller (Figure 5). AI has been deeply integrated into medical imaging technology, playing an increasingly important role in various aspects, ranging from image acquisition and processing to assisted reporting, follow-up planning, data storage, and data mining. Using modules like MONAI Label and TotalSegmentator on the platform, AI-based image automatic segmentation can be achieved, while the SlicerRadiomics module can extract parameters for omics analysis. Students can deeply experience the transformative power of AI technology in image processing and actively participate in clinical research work. The abundant online resources from the open-source community also provide strong support for conducting AI technology research using 3D Slicer. Students with programming skills can further develop and optimize the software according to their own needs or create specialized extensions to enhance their practical and innovative abilities. The application of the platform aligns with the integration of theory and practice in medical imaging education.

## 4. Discussion

The findings revealed that the use of 3D Slicer has influenced the field of medical image processing. Earlier, we noted that as technology advances, the academic community’s enthusiasm for incorporating 3D Slicer into medical imaging education will also increase. Table 3 encapsulates the current research trend regarding the impact of 3D Slicer on medical imaging education; this trend was derived from the findings of present study.

In recent years, multimedia has been a vital tool for educators and remains the predominant pedagogical approach [52,53]. Particularly in a field reliant on visual imagery, multimedia effectively engages multiple senses, stimulating student interest and playing an irreplaceable role [54,55]. However, with the ongoing advancement of computer technology and the rapid development of medical imaging, 2D images and non-interactive 3D images are no longer sufficient to meet the pedagogical demands of the modern era. Hands-on practice is increasingly becoming an integral component of instructional methodologies. This platform transforms displayed images and videos into interactive programs that students can manipulate directly, presenting content in a variety of formats, such as 2D, 3D, and partial tissue coverage [35]. This allows students to actively discover key concepts and progressively construct their own professional knowledge systems. Additionally, the platform can be integrated with emerging technologies such as augmented reality and virtual reality to establish virtual imaging laboratories [56].

Quantitative analysis and image segmentation, among various image analysis techniques, exhibit considerable utility in both clinical and research domains. Notably, Liao et al. [22] applied these methods for cranial surgery planning, while Cao et al. employed 3D Slicer for the volumetric analysis of intracranial hemorrhages [38]. Shi et al. also utilized 3D Slicer for preoperative assessments [43,45,47,50]. Additionally, Ari et al. [10] and Brown et al. [11] leveraged 3D Slicer for extracting and analyzing omics features. Despite the apparent distance from frontline teaching, it is imperative to recognize that a crucial facet of higher education is its application-oriented and employment-focused approach. Acquiring such pertinent skills during the graduate or undergraduate stages holds paramount significance for subsequent research endeavors and professional pursuits.

The incorporation of this platform into the teaching management process for medical imaging majors imbues students with a novel immersive experience, rendering learning content more clinically relevant. For educators, this necessitates a departure from traditional pedagogical methods dominated by explanation and presentation, instead stimulating student interest in autonomous learning through problem-based and project-based teaching approaches. This platform transcends the limitations of time and space, enabling students to conduct experiments and manipulate images and other content on their own computers, discovering and resolving various problems. Through such practices, students can more effectively engage in previewing and reviewing material, deepening their understanding of relevant knowledge, enhancing their practical application abilities, and elevating their professional proficiency.

However, 3D Slicer also has some limitations. Firstly, it has a steep learning curve, which may require beginners to invest time and effort to master its features. Secondly, the lack of an internationalized version creates a language barrier for users from non-English-speaking countries. Additionally, compatibility issues may arise as 3D Slicer was developed based on Insight Toolkit (ITK 5.6.0) and Visualization Toolkit (VTK 9.3.0), leading to potential conflicts with other software or operating systems. Lastly, 3D Slicer requires a certain level of computer hardware to handle large data or complex computations, which may lead to performance bottlenecks for users with inferior hardware configurations. The application of software platforms, as well as augmented reality and virtual reality, is still predominantly centered around pharmaceutical education and image interpretation. The integration of quantitative analysis functionalities with teaching methods is an area that urgently requires in-depth exploration. This platform’s actual effectiveness and value in education have not been fully verified. Future directions for the development of 3D Slicer include the following:

- The development of teaching methods and materials for medical image education based on 3D Slicer in order to improve the effectiveness and quality of medical image education.
- Simplifying the interface and operations of 3D Slicer to make it more user-friendly and accessible to more educational practitioners and students.
- Conducting educational experiments and evaluations to determine the effectiveness and feasibility of 3D Slicer in medical image education.
- Integration with other educational resources and tools. Medical imaging education requires the comprehensive use of various teaching resources and tools, such as textbooks, virtual laboratories, and simulators.
- The expansion of applications for the platform involves the further integration of imaging technology and diagnostic imaging needs, constituting a path for future research.
- Developing and expanding modules related to specific educational objectives to meet teaching requirements and enhance this software’s accessibility.

## 5. Conclusions

In conclusion, although 3D Slicer has demonstrated potential in medical image analysis, its application in medical image education remains limited and poses challenges. The transformation of teaching platforms constitutes a complex process requiring substantial and transformative changes. Achieving these changes will require collaboration among colleges, hospitals, educators, and relevant scientific professionals. Our literature review, which combines data from numerous studies, offers valuable insights into the application of 3D Slicer in medical imaging education, a dimension often neglected in academic settings. The advancement of artificial intelligence technology has rendered skills associated with it indispensable for professionals in the medical field. The 3D Slicer platform, encompassing a multitude of AI technologies, not only furnishes students with convenient tools for in-school learning but also lays a robust groundwork for their research and clinical endeavors. Its progressively escalating significance in medical imaging education will undoubtedly grow more manifest with the passage of time.

## Figures and Tables

**Table 1:** The main features of 3D Slicer.

Features	Introduction
Open source	Its source code is freely available and modifiable. This allows users to access, learn, and improve the software freely, catering to the needs of different users.
Multi-modal image processing	Handling various types of medical image data, including CT, MRI, PET, etc., it provides a range of powerful image-processing algorithms for tasks such as image segmentation, registration, reconstruction, etc.
Scalability	It offers a flexible plugin architecture that allows users to add new functionalities and algorithms as needed. This enables users to customize and extend the software’s capabilities according to their research requirements.
Visualization capabilities	It possesses rich visualization capabilities, such as volume rendering, surface rendering, slice display, etc., allowing medical image data to be presented in different ways. These capabilities enable users to better understand and analyze image data.
Research support	Providing a range of tools and functionalities for medical imaging research, it assists users in measurement, quantitative analysis, statistical modeling, etc., supporting the progress of medical research.
AI imaging analysis platform	It is a platform for a large number of AI imaging analysis technologies, not only reducing the threshold for application but also lowering the developmental difficulty, enhancing the practicality of AI technology.

**Table 2:** Exclusion and inclusion criteria.

Inclusion	Exclusion
Peer-reviewed articles.	Conference poster papers.
Research published in 2019 or later.	Studies not written in English.
Resources on higher education.	Redundant studies (i.e., studies with a similar focus conducted by the same author(s) and published in different venues, demonstrating little or no discernible difference; in our analysis, we specifically examined the most recent study conducted by the primary author).

**Table 4:** Comparison of teaching and platform-based teaching.

Traditional Teaching Drawbacks	Educational Platform Benefits
Limited methods, lacks variety	Boosts autonomy with diverse learning resources
Neglects student subjectivity	Enhances practical skills via virtual labs and scenarios
Confined to textbook content	Increases interest through interactive and immersive technologies
Monotonous methods hinder skill development	Reduces costs and is less reliant on physical resources

**Table 5:** Functions and applications of 3D Slicer.

Function	3D Slicer Application
Visualization and Analysis	Its various visualization methods, such as 3D reconstruction, slice display, and voxel rendering, enhance medical students’ understanding and analysis of imaging data for accurate report writing.
Measurement and Labeling	AI-driven measurement and labeling tools in 3D Slicer enable precise description and localization of lesions, structures, and anatomical features in reports, facilitating effective communication of observations and findings.
Interactivity and Usability	Its user-friendly interface and interactive operations make it easy for medical students to navigate and manipulate imaging data, improving efficiency and reducing report writing difficulty.
Data Integration and Sharing	Supporting various medical imaging data formats, including DICOM, NIfTI, and STL, 3D Slicer allows convenient integration and sharing of imaging data from different sources for comprehensive report writing.

## Data Availability

Data sharing is not applicable to this article as no datasets were generated or analyzed during the current study.
